# Enrollment and clients’ satisfaction with a community-based health insurance scheme: a community-based survey in Northwest Ethiopia

**DOI:** 10.1186/s12913-024-10570-7

**Published:** 2024-01-13

**Authors:** Ashenafi Kibret Sendekie, Ayenew Hailu Gebremichael, Melkamu Workie Tadesse

**Affiliations:** 1 Departement of Clinical Pharmacy, School of Pharmacy, College of Medicine and Health Sciences, University of Gondar, Gondar, Ethiopia; 2 Department of Sociology, School of Sociology and Social Work, College of Social Sciences and the Humanities, University of Gondar, Gondar, Ethiopia; 3 School of Economics, College of Business and Economics, University of Gondar, Gondar, Ethiopia

**Keywords:** Community-based health insurance, Enrollment, CBHI, Satisfaction, Ethiopia

## Abstract

**Background:**

Although the Ethiopian government has implemented a community-based health insurance (CBHI) program, community enrollment and clients’ satisfaction have not been well investigated in Gondar Zuria district, Northwest Ethiopia. This study assessed CBHI scheme enrollment, clients’ satisfaction, and associated factors among households in the district.

**Methods:**

A community-based cross-sectional survey assessed CBHI scheme enrollment and clients’ satisfaction among households in Gondar Zuria district, Northwest Ethiopia, from May to June 2022. A systematic random sampling method was used to select the study participants from eligible households. A home-to-home interview using a structured questionnaire was conducted. Data were analysed using the statistical packages for social sciences version 26. Logistic regression was used to identify variables associated with enrollment and clients’ satisfaction. A *p*-value < 0.05 was considered statistically significant.

**Results:**

Out of 410 participants, around two-thirds (64.9%) of the participants were enrolled in the CBHI scheme. Residency status (AOR = 1.38, 95% CI: 1.02–5.32; *p* = 0.038), time taken to reach a health facility (AOR = 1.01, 95% CI: 1.00–1.02; *p* = 0.001), and household size (AOR = 0.77, 95% CI: 0.67–0.88; *p* < 0.001) were significantly associated with CBHI scheme enrollment. Two-thirds (66.5%) of enrolled households were dissatisfied with the overall services provided; in particular, higher proportions were dissatisfied with the availability of medication and laboratory tests (88.7%). Household size (AOR = 1.31, 95% CI: 1.01–2.24; *p* = 0.043) and waiting time to get healthcare services (AOR = 3.14, 95% CI: 1.01–9.97; *p* = 0.047) were predictors of clients’ satisfaction with the CBHI scheme services.

**Conclusion:**

Although a promisingly high proportion of households were enrolled in the CBHI scheme, most of them were dissatisfied with the service. Improving waiting times to get health services, improving the availability of medications and laboratory tests, and other factors should be encouraged.

## Introduction

Health insurance continues to become one of the most important health financing programs and has been used as a complementary or substitute source of healthcare financing in the developing world [1]. Community-based health insurance (CBHI) schemes are often voluntary and applied by pooling funds from community members to protect themselves against the significant cost of pursuing medical care and treatment for medical conditions [2, 3]. Inability to afford out-of-pocket (OOP) expenditures has been one of the foremost obstacles to getting healthcare, especially for poor individuals and the vulnerable population at large [4]. Thus, CBHI has been implemented as a health financing tool and part of health transformation programs and policies in most low- and middle-income countries aimed at providing operational and proficient healthcare for citizens, most particularly for the poor and vulnerable [1, 5].

Though health insurance has been initiated in many countries, the majority of the population in developing countries suffers from financial devastation, and about 100 million are forced into poverty due to high OOP payments for healthcare services globally [6–8]. However, health insurance schemes in many low- and middle-income (LMIC) countries are still in their early stages of implementation, especially in most African countries, with the goal of universal coverage of the population in the health sector [9–12]. OOP payment to get health care is regressive as it limits admission to healthcare services for poor individuals, and it has contributed to the impoverishment of families due to having to pay for unexpected healthcare services at the time of illness [13].

There are many factors contributing to the enrollment and satisfaction of households with CBHI programs. Studies report that residency status, educational status, occupational status, household size, time taken to reach a health facility, and provided service qualities contribute to the enrollment of individuals or households in the healthcare services of CBHI [14–16]. On the other hand, the satisfaction of enrolled clients with the CBHI service was determined by the availability of medications and laboratory services, the qualification of healthcare service providers, and the waiting time to access health services [12, 17].

CBHI in Ethiopia is currently voluntary; individuals have the choice to enroll or not, and OOP has been a major means of financing health expenditures for several decades since the introduction of modern healthcare services [18, 19]. Nevertheless, recently, the Ethiopian government has developed a mutual health insurance strategy and a new policy for the CBHI schemes targeting employees from the rural and informal sectors through the Federal Ministry of Health (FMOH) of Ethiopia [20]. It has brought some improvements to the population’s health and the financing structure of healthcare [21]. The Ethiopian Health Insurance Service (EHIS) has been working to improve risk pooling among different groups of the population, such as between rich and poor, healthy and sick [22, 23]. CBHI packages in Ethiopia include all necessary family health services and curative care for disease conditions, which are part of the primary health packages, excluding dental and optical care and out-of-country referrals [22]. A high percentage of families registered in CBHI might be an indicator of the program's overall appeal and a gauge of how long it has been in place. More households will be able to ensure access to care when needed and avoid the financial burden of expensive treatments as more people join health insurance. However, in practice, CBHI frequently falls short of its potential, mainly due to low levels of involvement [24]. The commitment of authorities to support the program, and the attitude and awareness of the community towards the importance of health insurance services determine the enrollment and inclusion of individuals in the scheme [25–28]. Financial constraints and informal sector and economy dominance are also posing a problem of enrollment in the CBHI scheme because it is challenging to reach them through traditional enrollment channels. Subsidising premiums, information campaigns, and leveraging the informal sector are among the solutions.

Although EHIS’s initiatives have been practiced throughout the country and studies have been conducted regarding enrollment [16, 22] and clients’ satisfaction [12, 17, 29], the existing evidence focuses on either enrollment or clients' satisfaction in enrolled households. In addition, because of differences in community engagement with the CBHI scheme and variations in administrative and healthcare service provider facilities across different areas of the country, investigating the current study area is crucial to providing tailored insights because little is known about the extent of utilization of health insurance by households and the level of client satisfaction among CBHI scheme users in the study area. Furthermore, the clients’ level of utilization of and satisfaction with the CBHI scheme could be assessed in terms of individuals' perceptions of, expectations, and experiences with service delivery [30–32]. Consequently, the current study assessed the enrollment of households in CBHI, the level of clients’ satisfaction, and associated factors in Gondar Zuria, District, Northwest Ethiopia. The findings from the study may help to understand the extent of community involvement in CBHI and the level of satisfaction among individuals enrolled in the scheme. Besides, the findings serve as a baseline for further research and as input for improving the program outcomes by concerned authorities and stakeholders.

## Methods and materials

### Study design and setting

A community-based cross-sectional survey was conducted between April and June 2022 in Gondar Zuria District, Northwest Ethiopia. Gondar Zuria District is one of the twelve districts that constitute the Central Gondar Zone in Amhara National Regional State. The district has 44 *kebeles (*smallest administrative units) and shares a boundary with Lake Tana in the West, East Dembia in the North, Libo-Kemkem in the South and in the east with West Belesa. According to City and state facts report, the district has a population size of 231,866 in 2015 [33]. The district has been involved in CBHI service provision since 2018/19. According to the Gondar Zuria CBHI office, among the 43,965 eligible households in the study area, about 30,697 (69.8%) (25,231 payable and 5,466 indigent) households are enrolled in the CBHI scheme as of May 2022.

### Study population inclusion and exclusion criteria

The study participants included all household leads or household members responsible for providing information related to the household in Gondar Zuria District who had a verified household serial number and/or had an identification card that testified to their membership in the district. The data were collected from residents who were present around their homes during the data collection period and were willing to participate in the study. Individuals who were under 18 years old without parents or caretakers of the household were excluded from the survey.

### Sample size and sampling producers

The sample size was determined using the single population proportion formula with the assumption that the proportion of participants’ usage of CBHI and/or the level of satisfaction of 50% since no such study has been conducted so far in the area with a 95% confidence level. After considering 10% of the non-response rate, the final sample size was 422. Proportional allocation of the total sample size for each *kebele* in the study was demonstrated on the basis of their respective population size. Then, study participants from each *kebele* were recruited using systematic random sampling and coded according to their *kebele's* household record number (a number that can be found on the list of households in each *kebele*). A simple random sampling technique was also used to select the first participant to get the starting point. Thus, depending on the sampling interval, participants were selected until the required sample size was obtained.

### Operational definitions

#### Enrollment

Refers to the engagement of the community in CBHI. Here, participants are classified as “enrolled” or “not-enrolled” in the CBHI scheme [16].

#### Household

Refers to a person or a group of people related by blood, marriage, or adoption legally, who live together and share a common pot. Household size refers to the number of members of a household.

#### Level of clients’ satisfaction

Satisfaction refers to happiness with the way services in the CBHI scheme are arranged. “Level of client satisfaction” refers to the extent of satisfaction participants had in relation to the package of CBHI services. This was measured on a scale (ranging from 1 to 5) consisting of satisfaction measuring items. The tool has validated and has been used in the Ethiopian population. Each satisfaction-measuring item has five scales from “very dissatisfied” to “very satisfied,” with corresponding scores from 1 to 5, respectively. Then, the average score of responses from every item (minimum 1 and maximum 5) was transformed to provide a total satisfaction score ranging from 20 to 100% for each item used as a percentage mean score. “Dissatisfied” individuals were those who scored less than 75% on the satisfaction-measuring scale items, while those who had 75% and above scores on the scale were labelled as “satisfied” [29].

#### Kebele

Refers to a small governmental administrative unit in Ethiopia.

### Data collection instruments, producers and quality management

Data were collected via home-to-home interviews using structured questionnaires. The questionnaire was developed based on previous reports [9, 12, 20, 29, 34] and adapted in light of the local context and the research problem. The questionnaire was initially prepared in the English language and translated into Amharic, the local language, to make the data collection process smooth. It was pretested on 5% of the sample before the actual data collection in another adjacent district. Based on the pretesting exercise, the instrument was checked for completeness and consistency. In order to avoid repetition, the participants’ household record numbers were entered into Microsoft Excel 2016. On the field, the actual data collection was conducted by four trained individuals (training was given about the objectives of the study, the data collection producers, and ethical issues). A supervisor was recruited to oversee the data collection procedures and the data quality.

The level of clients’ satisfaction was measured using a scale consisting of seven items. The internal consistency of items was examined, and the reliability test resulted in a Cronbach’s alpha (α) of 0.88. Given the number of items, the value for Cronbach’s alpha (α) indicates the reliability of the instrument used.

### Data processing and analysis

After the data were collected, it was checked for completeness, consistency, and cleanliness, and then coded and entered into Epi Info version 8 and exported to the Statistical Package for Social Sciences (SPSS) version 26 for analysis. Statistical analysis on determinants of enrollment in the CBHI scheme and clients’ satisfaction with the services provided was performed independently. The normal distributions of the data were examined using a Q-Q plot and histogram. Continuous variables were presented using mean and standard deviation, and frequency and percentages were used to present the distribution of the categorical variables. Independent-samples t-test and one-way ANOVA tests were used to compare group mean scores on “level of client satisfaction” pertaining to services rendered in the CBHI scheme. Logistic regression analysis was also used to identify the association of variables with enrollment in the CBHI scheme and client satisfaction. A *p*-value < 0.05 was considered statistically significant.

## Results

### Socio-demographics characteristics of the study participants

Out of 422 approached study subjects, 410 participated in the study, resulting in a response rate of 97.2%. The majority of the study participants were males (76.1%) and rural residents (62%), with a mean (±SD) age of 46.4 (±12) years. Most of the study participants (266, 64.9%) used health insurance to cover their healthcare services (Table 1).

**Table 1:** Socio-demographic characteristics of the study participants at Gondar Zuria District, 2022 (*N*= 410)

**Socio-demographic variables**		**Frequency (%)**	**Mean (±SD)**
Sex	Male	312 (76.1)	
	Female	98(23.9)	
Age (in years)		-	46.4 (12)
Place of Residence	Rural	254(62)	
	Urban	156(38)	
Educational status	Unable to read or write	179(43.7)	
	Read and write only	65 (15.9)	
	Primary school	119 (29)	
	Secondary school	26(6.3)	
	College and university	21(5.1)	
Religious affiliation	Orthodox Christian	373 (91)	
	Muslim	37(9)	
Marital status	Single	44(10.7)	
	Married	310(75.6)	
	Divorced	34(8.3)	
	Widowed	22(5.4)	
Household size		-	5.4 (2.1)
Occupation	Farmer	259(63.2)	
	Merchant	64(15.6)	
	Daily labor	36(8.8)	
	Others	51(12.4)	
Coverage of healthcare cost	Out of pocket	130 (31.7)	
	Health insurance	266 (64.9)	
Average time taken from home to healthcare facility (in minutes)			46.4 (38.1)

### Enrollment of CBHI scheme and associated factors

Out of 410 study participants, around two-thirds (64.9%, 266) were enrolled in the CBHI scheme. Around two-thirds (64.9%) of the participants were enrolled in the CBHI scheme. Logistic regression analysis was performed to identify predictors of CBHI enrollment. Eventually, the multivariate logistic regression model showed that place of residence, family size, and average time taken from home to healthcare facility were significantly associated with enrollment in the CBHI scheme. After adjusting and other variables taken as constant, rural residents were found to be more likely to not be enrolled in the CBHI scheme compared to urban residents (AOR = 1.38, 95% CI: 1.02–5.32; *p* = 0.038). Similarly, participants who travelled for a longer time from home to healthcare facilities were found to be more likely to not get enrolled in the CBHI scheme (AOR = 1.01, 95% CI: 1.00–1.02; *p* = 0.001). In contrast, participants with a larger family size were found to be more likely to be enrolled in the CBHI scheme (AOR = 0.77, 95% CI: 0.67–0.88; *p* < 0.001) (Table 2).

**Table 2:** Association of independent variables with enrollment in CBHI

**Variables**		**Enrollment in CBHI scheme**		**95% CI**		***P*-value**
		**Not enrolled**	**Enrolled**	**COR**	**AOR**	
Sex	Male	114	198	1.31(0.80–2.13)	1.34(0.612–2.90)	0.463
	Female	30	68	1	1	
Age (in years) (mean ±SD)		43.8 (12.4)	47.8 (11.6)	0.92(0.85–0.99)	0.99(0.97–1.01)	0.270
Place of Residence	Rural	90	164	1.04(0.62–1.58)	1.38(1.02–5.32)	0.038*
	Urban	54	102	1	1	
Educational status	Unable to read or write	70	109	0.48(0.19–1.20)	0.64(0.22–1.86)	0.053
	Read and write only	16	49	0.25(0.09–0.69)	0.31(0.10–1.00)	
	Primary school	33	86	0.29(0.11–0.75)	0.36(0.12–1.02)	
	Secondary school	13	13	0.75(0.24–2.37)	0.61(0.18–2.08)	
	College and university	12	9	1	1	
Religious affiliation	Orthodox Christian	130	243	0.88(0.44–1.77)	0.66(0.29–1.47)	0.304
	Muslim	14	23	1	1	
Marital status	Single	14	30	2.10(0.60–7.37)	1.51(0.39–5.95)	0.429
	Married	113	197	2.58(0.85–7.82)	02.57(0.65–10.11)	
	Divorced	13	21	2.79(0.77–10.07)	2.25(0.57–8.92)	
	Widowed	4	18	1	1	
Household size (mean ±SD)		4.8 (1.9)	5.7 (2.1)	0.80(0.72–0.89)	0.77(0.67–0.88)	<0.001*
Occupation	Farmer	90	169	0.90(0.48–1.67)	0.89(0.20–3.94)	0.271
	Merchant	19	45	0.71(0.33–1.55)	0.77(0.31–1.88)	
	Daily labor	16	20	1.35(0.57–3.21)	1.96(0.71–5.41)	
	Others	19	32	1	1	
Average time taken from home to healthcare facility (minute) (mean ±SD)		55.6 (37.7)	41.4 (37.5)	1.34(1.07-2.71)	1.01(1.00–1.02)	0.001*

### Others clients’ satisfaction with the community-based health insurance scheme

**Fig. 1:**
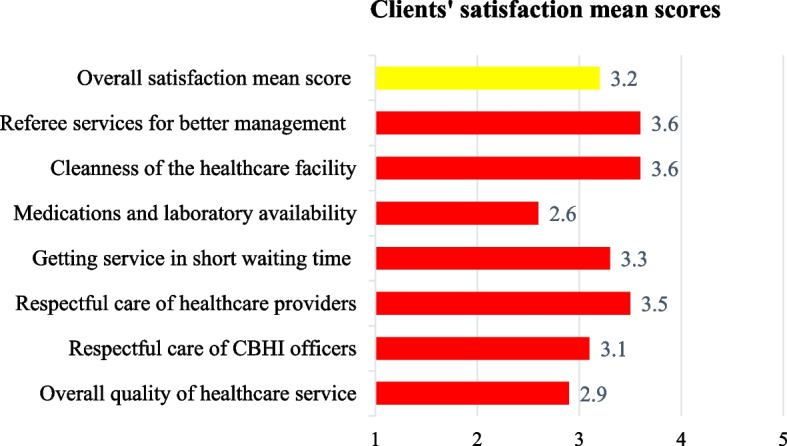
Satisfaction level of participants for each satisfaction measuring item

Overall, two-thirds (66.5%) of the enrolled households disclosed that they were dissatisfied with the CBHI scheme, with an overall mean satisfaction score of 3.2 (SD ±0.8) on a scale of 5 points. A significantly higher proportion of the study participants reported that they were not satisfied and/or very satisfied regarding the overall quality of healthcare service (68%) and the availability of medications and diagnostic laboratory tests (88.7%), with a significantly lower mean satisfaction score of 2.9 (SD±1.1) and 2.6 (SD±0.8), respectively. However, more than half of the participants were satisfied and/or very satisfied regarding the respectful care of healthcare providers, the cleanness of the healthcare facilities, and referee services. The mean (±SD) satisfaction scores were (3.5±1.2), (3.6±1.0), and (3.6 ±0.9), respectively, out of five points (Table 3 and Fig. 1).

**Table 3:** Level of client satisfaction on CBHI scheme service measuring items and overall satisfaction of clients (*N* = 266)

**Items**	**Satisfaction level (%)**				
	**Very dissatisfied**	**Dissatisfied**	**Neutral**	**Satisfied**	**Very satisfied**
Overall quality of healthcare service	44 (16.5)	44 (16.5)	93 (35)	74(27.8)	11 (4.2)
Respectful care of CBHI SCHEME officers	18 (6.8)	68 (25.6)	77 (28.9)	81 (30.5)	22 (8.3)
Respectful care of healthcare providers	9 (3.4)	59 (22.2)	58 (21.8)	68 (25.6)	72 (27.1)
Getting services in a short waiting time	6 (2.3)	71 (26.7)	70 (26.3)	75 (28.2)	44 (16.5)
Medications and laboratory availability	26 (9.8)	87 (32.7)	123 (46.2)	28 (10.5)	2 (0.8)
Cleanness of the healthcare facility	1 (0.4)	49 (18.4)	72 (27.1)	86 (32.3)	58 (21.8)
Referee services for better management	1 (0.4)	30 (11.3)	86 (32.3)	96 (36.1)	53 (19.9)
**Overall satisfaction level**			**Frequency (%)**		
	Dissatisfied		177 (66.5)		
	Satisfied		89 (33.5)		

^*^Indicates *p* < 0.05, other includes, students; homemakers

### Satisfaction differences among different groups of the participants

The independent samples t-test showed that there was a significant satisfaction difference between different household sizes (t = 1.7; *p* = 0.044). Participants who had less than or equal to five household members had a significantly better satisfaction score (Mn = 3.5) than those with more than five household members (Mn = 3.1).

A one-way ANOVA test also indicated that there were significant differences in the mean satisfaction scores of participants with different educational status (F = 3.5; *p* = 0.008) and occupations (F = 5.9; *p* = 0.001) (Table 4). The Tukey post hoc test also revealed that college and university-graduated participants had significantly higher satisfaction mean scores (Mn = 3.9) than those who were unable to read and write (Mn = 3.2); *p* = 0.048; and who could read and write only (Mn = 3.0); *p* = 0.012. Regarding the occupation of study participants, merchants had significantly higher satisfaction mean scores (Mn = 3.4) than farmers (Mn = 3.1); *p* = 0.045. Similarly, daily laborers had significantly higher satisfaction mean scores (Mn = 3.7) than farmers (Mn = 3.1); *p* = 0.001.

**Table 4:** Mean satisfaction score differences among the respondents regarding CBHI scheme services are illustrated with the Independent-samples T-test and One-Way ANOVA analysis

**Variables**	**Category**	**Satisfaction score of the CBHI scheme services**		
		**Mean (±SD)**	**T/F**	***P*-value**
Sex	Male	3.2(0.8)	0.4^a^	0.669
	Female	3.2(0.9)		
Age (in years)	< 50	3.2 (0.8)	-0.7^a^	0.459
	≥ 50	3.3 (0.8)		
Place of Residence	Rural	3.2(0.8)	-1.1*	0.264
	Urban	3.3(0.9)		
Religious affiliation	Orthodox Christian	3.2 (0.8)	-1.1^a^	0.280
	Muslim	3.4(0.8)		
Marital status	Single	3.5(0.8)	1.8^b^	0.144
	Married	3.2 (0.8)		
	Divorced	3.2 (0.8)		
	Widowed	3.4 (0.7)		
Household size	≤ 5	3.5 (0.8)	1.7^a^	**0.044**
	> 5	3.1 (0.8)		
Educational status	Unable to read or write	3.2(0.8)	3.5^b^	**0.008**
	Read and write only	3.0 (0.9)		
	Primary education	3.4 (0.8)		
	Secondary education	3.2(0.8)		
	College and university	3.9(0.7)		
Occupation	Farmer	3.1 (0.8)	5.9^b^	**0.001**
	Merchant	3.4 (0.8)		
	Daily labor	3.7 (0.7)		
	Others	3.2 (0.7)		
Average-waiting time to get healthcare services (minute)	≤ 50	3.3 (0.9)	1.41^a^	0.159
	> 50	3.2 (0.8)		

^a^T**-**Independent-samples T-test

^b^F-one-way ANOVA test; age, household size and waiting time to get healthcare services was transformed into categorical variables from their average values

Bold values denote significant differences (*p* <0.05)

### Associated factors of clients’ satisfaction in community-based health insurance schemes

Crude logistic regression analysis showed that there are important factors linked to the level of client satisfaction. Educational status, marital status, household size, occupational status, and waiting time to get healthcare services at the facilities were significantly associated with clients’ satisfaction level in the crude logistic regression. However, the multivariable logistic regression model showed that only household size and waiting time at health facilities to get healthcare access were significantly associated with clients’ satisfaction with the CBHI scheme services.

After adjusting and controlling other variables, participants who had ≤ 5 household members were found to be more likely to have satisfaction with the CBHI scheme services compared with those having less than five members (AOR = 1.31, 95% CI: 1.01–2.24; *p* = 0.043). Similarly, participants who waited less than 50 minutes to get healthcare access at the health facility were more likely to be satisfied compared with participants who were waiting longer than 50 minutes (AOR = 3.14, 95% CI: 1.01–9.97; *p* = 0.047) (Table 5).

**Table 5:** Association of independent variables with clients’ satisfaction on CBHI scheme healthcare services

**Variables**		**Clients’ satisfaction**		**95% CI**		***P*-value**
		**Satisfied**	**Dissatisfied**	**COR**	**AOR**	
Sex	Male	64	131	0.93(0.51-1.69)	1.41(0.6-3.32)	0.43
	Female	22	42	1	1	
Age (in years)	< 50	42	95	0.77(0.463-1.285)	0.75(0.42-1.32)	0.315
	≥ 50	47	82	1	1	
Place of Residence	Rural	48	115	0.63(0.38-1.06)	2.49(0.70-8.88)	0.16
	Urban	41	62	1	1	
Educational status	Unable to read or write	33	79	0.33(0.08-1.32)	0.35(0.07-1.87)	0.553
	Read and write only	13	33	0.32(0.07-1.36)	0.38(0.06-2.24)	
	Primary school	34	52	0.52(0.13-2.09)	0.57(0.12-2.99)	
	Secondary school	4	9	0.36(0.06-2.08)	0.49(0.07-3.60)	
	College and university	5	4	1	1	
Religious affiliation	Orthodox Christian	79	164	0.63(0.26-1.49)	0.70(0.25-1.94)	0.487
	Muslim	10	13	1	1	
Marital status	Single	20	20	1.13(0.36-3.51)	0.81(0.22-3.04)	0.064
	Married	52	129	0.45(0.17-1.24)	0.38(0.10-1.06)	
	Divorced	9	19	0.53(0.15-1.84)	0.40(0.10-1.54)	
	Widowed	8	9	1	1	
Household size	≤ 5	90	44	1.06(0.64-1.76)	1.31(1.01 -2.24)	0.043*
	> 5	87	45	1	1	
Occupation	Farmer	35	102	0.87(0.41-1.84)	0.99(0.40-2.46)	0.117
	Merchant	24	30	2.03(0.88-4.69)	2.07(0.76-5.44)	
	Daily labor	17	12	3.60(1.35-9.57)	2.71(0.83-8.86)	
	Others	13	33	1	1	
Waiting time to get healthcare services (minute)	≤ 50	48	68	1.88(1.121-3.141)	3.14(1.01-9.79)	0.047*
	> 50	41	109	1	1	

*CI* Confidence interval, *COR* Crude odds ratio, *AOR*, Adjusted odds ratio; age, household size and waiting time to get healthcare service were transformed into categorized variables from their average values; * indicates *p* < 0.05

## Discussion

This community-based survey aimed to assess the enrollment of households in the CBHI scheme and the level of client satisfaction with the services provided by the program. Consequently, this study showed that most participants were enrolled in the CBHI scheme, and two-thirds (66.5%) of the enrolled participants were dissatisfied with the services of the program. Residency status, time taken to health facilities, and household size were factors associated with the enrollment of the CHBI scheme, while household size and waiting time to get healthcare services were variables significantly associated with enrolled clients’ level of satisfaction with the CBHI scheme.

The current study has shown that around two-thirds of the participants were enrolled in the CBHI scheme. This figure indicates a better enrollment rate than the report by EHIS, which indicated a national rate of 58% [20]. This implies the existence of factors that motivate residents in the study area to enroll in the program. However, it could still be encouraged to engage the communities in the program. It has an important implication for healthcare financing by protecting citizens from catastrophic health expenditures. Consistent with previous studies [14, 15], the current findings revealed that residence, household size, and time taken from home to healthcare facility were predictor variables for CBHI scheme enrollment among households. Consequently, rural residents and participants who travelled a longer distance from home to healthcare facilities were found to have lower enrollment in the CBHI scheme compared with urban residents and participants who travelled a shorter distance, respectively. This might be because of lower awareness, poor knowledge regarding the programs and services, and a lack of information about how they engage with the health insurance systems. Rural residents and those who live at a longer distance from health facilities may not have better knowledge and access to information about health insurance systems and services. This intern decreases their enrollment in the insurance scheme. Therefore, rural communities living a longer distance from home to health facilities may need attention. The service providers must design policies or evaluate their implementation to better accommodate rural communities. This study also demonstrated that participants with a larger family size were more inclined to join in the CBHI scheme. This could be the case because people with larger families might not be able to afford the OOP expenses associated with receiving healthcare. As a result, individuals are free to select health insurance plans, enlist in the program, and go via the CBHI for only the best pre-paid coverage.

This study revealed that, despite the fact that most of the community had enrolled in the CBHI, a lower proportion (33.5%) of the clients were satisfied with the services of the program. This finding is much lower than the previous studies [9, 12, 35, 36]. The finding implies that the CBHI scheme office may not go through the way it is designed to because of different reasons. This can compromise the service of the program and lead to clients’ dissatisfaction. For instance, in this study, a significantly higher proportion of participants were not satisfied with the overall quality of services, and more than 80% of the participants were dissatisfied with the availability of medications and laboratory tests. This may contribute to the overall dissatisfaction of the clients. The clients also reported that they were forced to leave refereed health facilities without receiving healthcare access because of the inability of the district to recover the costs from the previous one and two years; this in turn affects the clients’ satisfaction with the overall services. However, more than half of the participants reported that they were satisfied and/or very satisfied with the respectful care of healthcare providers, the cleanliness of the healthcare facilities, and the referee services when the program recovered the costs for healthcare facilities. The finding may suggest that the health insurance system should adhere to the interests of clients in its implementation.

In this study, satisfaction levels among different participants were significantly different. Thus, the findings showed that there was a significant satisfaction difference between different household size members, different waiting times to get healthcare services, different educational statuses, and different occupational types. Consequently, participants with larger household sizes were found to have significantly lower satisfaction scores compared to those with lower household family members. This might be partially justified because clients with larger family sizes could pay a higher pre-paid cost of coverage proportionally to their household size, which in turn affects their perceptions and satisfaction with the services of the program. The association of household size and clients’ satisfaction in this study also showed that participants who had smaller household sizes were found to be more likely to have higher satisfaction with the CBHI scheme services compared to those with larger household families. This might be because households with large families might be reluctant to pay for their pre-paid cost coverage based on their family size. This can contribute to a lower perceived service quality. Thus, the compromised healthcare service may make them dissatisfied, in contrast to their expectations.

This study also revealed that participants who waited a shorter time to get healthcare access at the health facility had a high satisfaction score. An impact of waiting time on the satisfaction level also uncovered that patients who were waiting a shorter time were found to be more likely to be satisfied compared with participants who were awaiting a longer time to get healthcare services from health facilities. This finding agrees with a previous study conducted by Haile et al [12]. The finding indicates that healthcare providers should be encouraged to provide healthcare for the clients as early as possible on their referral or admission to the health facility.

There is also a significant satisfaction mean score difference among different participants regarding their educational status and occupational types. The finding revealed that college and university-graduated participants had significantly higher satisfaction mean scores compared to participants who were unable to read and write and who could read and write. This could be because those participants with higher educational levels may have better awareness and knowledge regarding how the services of the program can be implemented, making them more likely to adhere to the services that can result in high satisfaction scores compared to participants with lower educational status. Additionally, in terms of occupational characteristics of study participants, merchants had significantly higher satisfaction mean scores than farmers on CBHI scheme services. This might be related to their level of awareness, knowledge, and information regarding the service in general, which can affect their level of involvement and their level of satisfaction with the service. However, this study did not show a statistically significant association between the educational status and occupation of participants and their level of satisfaction.

Generally, the study highlighted the level of household engagement with the CBHI scheme and clients’ satisfaction in Northwest Ethiopia. The findings showed that the services provided by the program should be encouraged. The ultimate goal for which every health system should strive is to achieve and maintain client satisfaction and increase the provision of service to customers. Therefore, the commitment of the district authorities to support the scheme through the implementation of cost recovery would have a great value in improving clients’ satisfaction. Additionally, healthcare facilities could implement the CBHI services based on the aim, which is to maintain the healthcare financial balance of individuals and the community at large.

### Strength and limitation of the study

This study presents a comprehensive finding of enrollment and satisfaction of enrolled households with the CBHI scheme and associated factors in the study area. This is the first study to assess both enrollment and satisfaction of clients. However, this study has some limitations. Firstly, this study captures data at a single point in time, making it impossible to determine the cause-and-effect relationship between variables. Secondly, participants may not accurately recall their past experiences, particularly regarding sensitive topics like healthcare utilization, quality of services, and other financial information. This can lead to biassed results. Furthermore, the survey relies on participants to accurately report their own experiences and opinions. This can be problematic due to social desirability bias, where participants may under-report healthcare services because they need further improvements. Lastly, findings from a single woreda may not be generalizable to other communities with different contexts and characteristics. Therefore, the authors welcome future research using triangulation, combining cross-sectional surveys with qualitative methods, and prospective longitudinal studies to track participants over time and assess changes in enrolment, satisfaction, and other factors.

## Conclusion

This study concluded that a significant proportion of households in the district were enrolled in the CBHI scheme, but most of these participants were dissatisfied with the program's services. Significantly, a higher proportion of participants were not satisfied with the overall quality of services and/or the availability of medications and laboratory tests. Household family size and waiting time to get healthcare access were predictors clients’ satisfaction with the CBHI scheme services. Therefore, the CBHI services need to be monitored and audited based on its objectives, in particular the overall quality of service and availability of medications, and laboratory tests should need special attentions. 

## Data Availability

The datasets generated and/or analyzed during the current study are not publicly available to protect from unnecessary abuse of full data of the participants, but are available from the corresponding author on reasonable request.

## Abbreviations

CBHI: Community-based health insurance
EHIS: Ethiopian health insurance service
FMOH: Federal Ministry of health
OOP: Out-Of-pocket
